# Genetic Signature of Anthropogenic Population Collapse in Orang-utans

**DOI:** 10.1371/journal.pbio.0040025

**Published:** 2006-01-24

**Authors:** Benoît Goossens, Lounès Chikhi, Marc Ancrenaz, Isabelle Lackman-Ancrenaz, Patrick Andau, Michael W Bruford

**Affiliations:** **1**Biodiversity and Ecological Processes Group, Cardiff School of Biosciences, Cardiff University, Cardiff, United Kingdom; **2**UMR 5174 Evolution et Diversité Biologique, Université Paul Sabatier, Toulouse, France; **3**Kinabatangan Orang-utan Conservation Project, Sandakan, Sabah, Malaysia; **4**ITBC, Universiti Malaysia Sabah, Kota Kinabalu, Sabah, Malaysia; **5**Pittsburgh Zoo, Pittsburgh, Pennsylvania, United States of America; **6**Sabah Wildlife Department, Wisma Muis, Kota Kinabalu, Sabah, Malaysia; Zoological Society of LondonUnited Kingdom

## Abstract

Great ape populations are undergoing a dramatic decline, which is predicted to result in their extinction in the wild from entire regions in the near future. Recent findings have particularly focused on African apes, and have implicated multiple factors contributing to this decline, such as deforestation, hunting, and disease. Less well-publicised, but equally dramatic, has been the decline in orang-utans, whose distribution is limited to parts of Sumatra and Borneo. Using the largest-ever genetic sample from wild orang-utan populations, we show strong evidence for a recent demographic collapse in North Eastern Borneo and demonstrate that this signature is independent of the mutation and demographic models used. This is the first demonstration that genetic data can detect and quantify the effect of recent, human-induced deforestation and habitat fragmentation on an endangered species. Because current demographic collapses are usually confounded by ancient events, this suggests a much more dramatic decline than demographic data alone and emphasises the need for major conservation efforts.

## Abstract 2

A genetic analysis reveals a recent and dramatic decline of orangutan populations in Malaysia, and demonstrates that genetic data can quantify the effects of recent anthropogenic changes on endangered species.

## Introduction

The orang-utan is the only great ape found outside Africa [1,2]. Due to its large home range, semi-solitary nature, arboreal lifestyle, and reluctance to cross open areas, it is probably the species most affected by the increasing fragmentation of tropical forests [1,2]. Despite a wide Pleistocene distribution in South-East Asia, including Sumatra, Borneo, Java, and mainland Asia [2], wild orang-utan populations are only found in Northern Sumatra and Borneo today (*Pongo abelii,* and P. pygmaeus, respectively). On these two islands they survive mainly in fragmented and isolated populations, threatened by illegal hunting, the pet trade, and habitat loss, fragmentation, and degradation as a consequence of harvesting and the post-logging conversion of forested areas into oil palm plantations [1–4]. In the Leuser area (Sumatra) alone, it is estimated that 1,000 orang-utans have been killed every year between 1993 and 2000 out of the approximately 12,000 present in the early 1990s [3–5]. In Borneo, the population is estimated to have dropped by 33% between 1996 and 1997 due to drought and fires [1,2]. Across the whole range, it is generally estimated that numbers of orang-utans have experienced a 10-fold decrease from approximately 315,000 around 1900 to approximately 27,000 in 1997 ([1,2], but see Discussion below). However, conservation management is currently hampered because a number of uncertainties remain concerning (1) the original size estimates of the orang-utan population, (2) the trajectory of the recent decline, and (3) its causes. Estimates by Payne [6] restricted to Sabah suggested that more than 20,000 orang-utans were still present in the 1980s. These estimates have been challenged as being overoptimistic by at least an order of magnitude [1]. However, if correct, they would indicate that orang-utans were much more numerous than previously thought only a few decades ago and that the ongoing decline in orang-utans is more recent and drastic than previously believed. It has also been suggested that the recent decline of orang-utan populations represents only part of a general trend that started with the arrival of the first hunter-gatherers in South-East Asia, some 40,000 y ago. The later arrival of the first farmers 5,000 y ago [7] may also have influenced ancient populations of orang-utans. This effect is however difficult to evaluate. Farmers certainly did not clear the forest on a scale comparable to that which occurred during the last century [8], but they could have significantly impacted on orang-utan populations through hunting. Indeed, a number of recent studies have demonstrated that “overkill” scenarios whereby farmers or hunter-gatherers, arriving in newly colonised areas, were responsible for the collapse and extinction of a number of vertebrates, with a larger effect on large mammals, in America [9,10], Madagascar [11], or South-East Asia [10]. The impact seems to have been larger in smaller islands (e.g., [12]), which explains why orang-utans and elephants are still present in Borneo. Climatic changes have also played a role in shaping the distribution of South-East Asian mammals [13], including orang-utans, but it is not clear whether these changes have left a mark on the genetic makeup of the remaining populations. For instance, using mitochondrial DNA data, Warren et al. [14] found much higher levels of genetic diversity in orang-utans from Borneo than in humans and East African chimpanzees. Warren et al. hence argued that Bornean orang-utans have not experienced a serious genetic bottleneck. However, it is certain that all the preceding factors have played a role in the history of remnant orang-utan populations. Dating the time at which populations started to decrease would thus be crucial in elucidating the relative effects of climatic events, hunting, either Prehistoric or by farmers, and the more recent forest fragmentation.

Although the importance of genetic diversity for the fitness and long-term survival of species is increasingly clear [15–17], the effects of very recent anthropogenic population fragmentation on genetic diversity are typically difficult to demonstrate [18,19]. Indeed, the genetic structure of most species is dominated by ancient demographic events such as population bottlenecks and/or expansions following climatic changes [18–22], which confound the signature of contemporary demographic collapse. Even in cases such as the recent near-extinction by humans of the northern hairy-nosed wombat in Australia [23], detailed statistical analysis of genetic diversity patterns has shown that the population decline occurred or started before human activities [18]: The only signature visible in the genetic data pertained to events predating the observed and well-documented population decline (see below). Thus, although the future of wild orang-utan populations seems bleak, and the impact of human activities on the environment well documented [4,8,24], the impact of recent environment changes on the genetic diversity of orang-utans is far from clear. Improvements in statistical modelling of stochastic systems have allowed the development of computational model-based Bayesian methods [19,25]. Such methods provide a solution to the previous limitations by making full use of the information present in the genetic data, provided that recent demographic events have been substantial enough.

We studied orang-utan populations in the Lower Kinabatangan floodplain in Eastern Sabah, a region that has experienced large-scale commercial timber exploitation and agriculture since the mid-1950s [8]. Faecal and hair samples were collected from wild orang-utans during boat surveys along the Kinabatangan River or during line transects to estimate nest densities [26]. Two hundred different animals were genetically identified using 14 microsatellites.

Three different but complementary approaches were used to detect, quantify, and date the decline in orang-utan populations. The first approach is based on summaries of the allelic frequency spectrum [27,28] and was used to detect either a population expansion or decline. It relies on the loss or excess of rare alleles expected in bottlenecked or expanding populations, respectively, and uses simulations under different mutation models to detect departures from mutation-drift equilibrium. Under this approach, to which we refer as the *EWCL* approach (for Ewens, Watterson, Cornuet, and Luikart; see Materials and Methods), population size change cannot be dated or quantified. This method was applied to the whole Kinabatangan dataset (nine sampling units, *n* = 200 individuals, 14 loci). The two other approaches used were Bayesian methods using the information from the full allelic distributions and shall be referred to as the *Beaumont* [18] and the *Storz and Beaumont* [21] method. The *Beaumont* method assumes a model of population size change from *N_1_* (in the past) to *N_0_* (at the time of sampling). This change can be linear or exponential, and the ratio *N_0_*/*N_1_* can be inferred. The population size change can thus be quantified, but it cannot be dated. The *Storz and Beaumont* method [21] allows the estimation of *N_0_* and *N_1_,* rather than their ratio, and, crucially, also *T,* the time (in generations) since the population changed. The two Bayesian methods were applied to two subsets of the data for computational reasons.

## Results

### Detection of a Population Size Change

The results of the *EWCL* approach indicate that, regardless of the mutation model assumed, all nine samples exhibit a strong and significant signal for a population bottleneck, through the loss of rare alleles (Table S1). Given that the 200 individuals sampled represent approximately 20% of the total Kinabatangan population [26], this is evidence for a general population decline across the whole area sampled (40,000 ha with 27,000 ha for the Sanctuary). However, this approach cannot quantify or date the population bottleneck.

### Quantification of the Population Size Change

**Figure 1:**
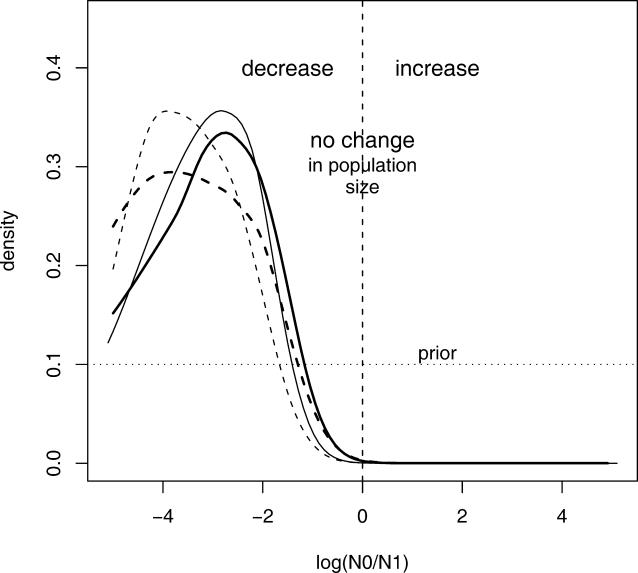
Population Size Change Solid curves correspond to the posterior distributions under a model of exponential population size change. Dashed curves were obtained under a model of linear change. Thin and thick lines correspond to results obtained for populations S1 and S2, respectively (see main text for details). *r* = *N_0_*/*N_1_* represents the ratio of present *(N_0_)* to past *(N_1_)* population size. Whichever demographic model or population is used, there is no support for positive values (increase in population size) or values close to zero (no significant change in population size). The prior distribution is shown for comparison (flat dotted line).

The quantification is achieved using the *Beaumont* method, which allows us to estimate the posterior densities for log*(N_0_*/*N_1_)*, shown in Figure 1 together with the flat prior (dotted line), for comparison. This figure indicates that the present-day genetic structure of orang-utans is strongly influenced by a dramatic decrease in population size, with no support for growing or even stable populations, confirming the *EWCL* analysis. This result is independent of the demographic model (exponential versus linear) and the population analysed (S1 versus S2). Regardless of the model and population analysed, the 90% and 95% quantiles for log*(N_0_*/*N_1_)* suggest that orang-utan populations have decreased by at least a factor of approximately 100 and approximately 50, respectively. The full posterior distribution actually shows that the population collapse may have been by more than two orders of magnitude, whichever population and model of population decline is assumed. However, this analysis does not allow us to estimate present and past population sizes (only their ratio), but this is done using the *Storz and Beaumont* approach.

### Quantification of Past and Present Population Sizes

**Figure 2:**
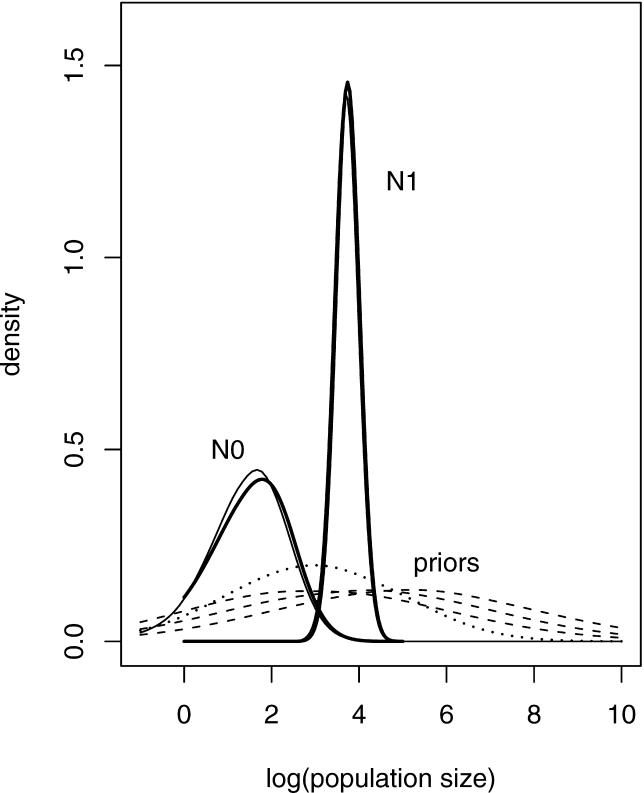
Ancestral and Present Population Sizes The posterior distributions are represented in a logarithmic scale and show very little overlap, confirming that *N_0_* is much smaller than *N_1_*. This result holds for the two populations analysed. The thin and thick lines correspond to S1 and S2, respectively. The priors are also shown for *N_0_* (dotted line) and *N_1_* (dashed line).

The *Storz and Beaumont* approach shows consistently that the posterior distributions for log*(N_0_)* and log*(N_1_),* have very limited overlap with medians of approximately 30 and approximately 5000, for *N_0_* and *N_1_,* respectively (Figure 2), again confirming the two previous approaches. The posterior distributions are also very different from the priors (Figure 2, dashed and dotted lines) and indistinguishable for S1 and S2 whichever prior is used for both *N_1_* and *N_0_,* indicating that the genetic signature for a population collapse is very strong and of the same magnitude on both sides of the Kinabatangan river.

### Dating the Population Collapse

**Figure 3:**
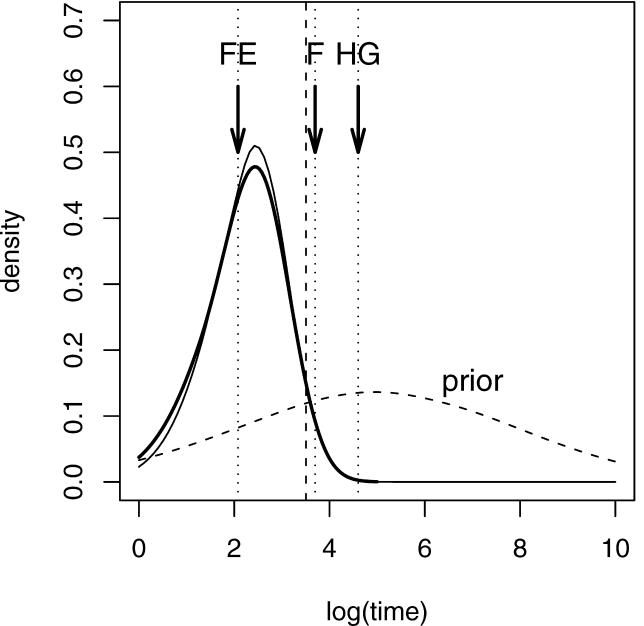
Time since the Population Collapse The posterior distribution for the time since the population collapse is represented on a logarithmic scale. These distributions have a median around 210 y. Most of their mass is concentrated in recent years with a sharp decrease as time goes back. Indeed, 10%, 20%, 50%, 80%, and 90% of the distribution mass are below 10, 35, 210, 950, and 1,900 y, respectively. The thin and thick lines correspond to S1 and S2, respectively. The prior is shown as a dashed line, its median being 100,000 y ago (see text). The vertical dashed line corresponds to the 95% quantile of the posterior distribution. Arrows correspond to the dates of arrival of the first hunter-gatherers (HG) or farmers (F), or to the start of the forest exploitation (FE).

**Figure 4:**
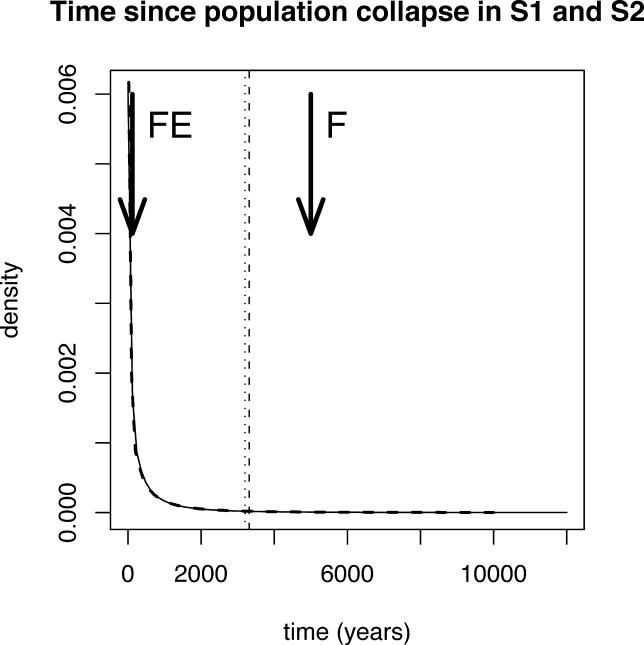
Time since the Population Collapse (in Years) The two curves represent the posterior distributions for S1 (solid line) and S2 (dashed line) in a natural rather than a logarithmic scale. The two posterior distributions are nearly indistinguishable and clearly show that the orang-utan population collapse most likely started in the last decades. The vertical dashed and dotted lines correspond to the 95% quantile of the posterior distributions for S1 and S2, respectively. The arrows correspond to the dates of arrival of the first farmers (F) or to the start of the forest exploitation (FE). The arrival of the first hunter-gatherers would be far outside this figure on the right, some 40,000 y ago.

The *Storz and Beaumont* approach also allows us to date the collapse by providing a posterior distribution for *T,* the time (in generations) at which the population started to decrease. This distribution has a median of approximately 210 y, despite the fact that, in the prior, the median, mean, and mode were 100,000 y ago, that is, 500 times older (Figure 3). This figure also shows that more than a third of the posterior distribution coincides with the last century and the modal value (in natural rather than logarithmic scale) in the most recent decades (Figure 4). Given that the exploitation of Sabah's forests started in 1890 and accelerated first in the 1950s and again in the 1970s [8], this distribution is rather informative. Its shape shows that the posterior probability for *T* decreases very quickly further back in time (Figure 4), whereas we took a prior in which this probability was increasing. To quantify this difference between the prior and the posterior, it is worth noting that dates older than 10,000 y ago had a prior support of approximately 62%, whereas they only have a posterior support of 1.4%. Clearly, these results suggest that Prehistoric hunting and Pleistocene climatic events cannot reasonably explain the detected genetic signature for a population decrease. Similarly, the arrival of the first farmers does not appear to be a very likely explanation of the decrease, as Figures 3 and 4 show.

## Discussion

Our results show that the orang-utan populations have decreased by more than 95% in the last centuries, and that this decline is likely to have taken place even more recently. Importantly, it is clear that the conclusions are robust, because the parameters inputted into the models did not affect the outcome. For example, orang-utan generation time here was modelled at 8 y, as current field observations indicate in the Kinabatangan. However, even if we double or triple this value, the results do not change qualitatively. For instance, if we double the generation time, the support for dates older than 10,000 y ago would only increase to approximately 3% (despite a prior of 62%). Indeed, the posterior support for Prehistoric hunting or an older climate-driven decrease is still minute compared to the support for events taking place in the last centuries, despite the much higher support we gave to old events in the choice of the priors (Figures 3 and 4).

The only major event that may have significantly influenced orang-utan populations in the last decades or centuries is the recent anthropogenic fragmentation of the habitat. Even the arrival of the first farmers some 5,000 y ago [7] cannot account for our results, because the support for dates older than 5,000 y would only increase to 7% (against a prior of 66%) if we doubled the generation time. To put our results into perspective, it is worth noting that when Beaumont [18] re-analysed data from 16 microsatellite loci from 28 northern hairy-nosed wombats, known to have sharply decreased from thousands of individuals in the last 120 y to less than 30 in the 1980s [23], he found that the population had been declining over a much longer period than the last 120 years. Similarly, Storz et al. [22] also found a significant signal for population contraction in savannah baboons, but were unable to date it to recent anthropogenic changes. The most probable dates were between 1,000 and 250,000 y ago, and were hence more compatible with climatic events. Thus, the fact that our results display posterior *T* distributions having their mode in the very last decades, not in the last centuries, leads to the conclusion that the signal we pick is of a very recent decrease, and a consequence of the recent fragmentation of the environment.

The results are also sensitive to the mutation processes assumed for the microsatellite markers used. Three different mutation models were first used to test the effect of the mutation model on our results. As Table S1 shows, the signal is consistent across mutation models for all subpopulations. Indeed, no locus appeared to exhibit a significantly higher than average level of significance, as could be shown using a randomization test. Moreover, the lowest support was obtained for the single stepwise mutation model. Since this is the mutation model assumed by the two Bayesian approaches, the results here are conservative. Moreover, this is the mutation model for which the signal is least significant. For the demographic model, we tested the consistency of the inference by using both linear and exponential changes. The results were virtually identical in the two cases. One potential caveat is the assumption that there is no significant substructure within the samples analysed. Indeed, population structure, if important, can generate genealogies that resemble those obtained under a population collapse [19]. In practice, one must ask whether it is reasonable to assume that population structure prior to habitat fragmentation was strong enough to generate the signal of an apparent three-order magnitude population crash. Indeed, our results indicate that the median for *r,* the ratio of the ancient to current population size, is on the order of 10^3^. Although it is difficult to determine the amount of population structure that could mimic a bottleneck of that intensity, it seems reasonable to assume that an average *F*_ST_ lower than, say, 0.05 would be unlikely to generate this kind of population crash signal. Otherwise, most populations across many species would exhibit signals of population crashes across loci. In other words, for population structure to be responsible for this signal would require that ancient orang-utan populations be genetically highly differentiated. This is contradicted by the very low *F*_ST_ values observed between samples from the same riverside (pairwise *F*_ST_ values were less than ~0.04 with an average of ~0.025) [29]. This is also at odds with estimates of gene flow that we obtained for the remnant forest fragments [29]. Indeed, using a method that allows the estimation of recent gene flow in sets of populations [30], we were able to determine that gene flow is the rule rather than the exception between forest fragments from the same side of the river, despite the fragmentation. To confirm this we applied a method that attempts to separate the effects of genetic drift and gene flow at equilibrium [31], to estimate the amount of population substructure. This analysis confirms that population structure is very limited with an average *F* of 0.047 across populations (Table S2). It is important to note that this method estimates the amount of drift within populations, based on the assumption that equilibrium is reached. Given that populations have decreased and are now fragmented, the estimates obtained are overestimates of the level of isolation of the populations prior to the population crash. Given the consistency of three different methods to indicate low levels of genetic differentiation between forest fragments, it seems very unlikely that population structure within these fragments would be so high as to mimic a population crash as dramatic as the one demonstrated here. Finally, from a biological point of view, strong population structure does not seem a very realistic assumption in pristine Borneo because habitats were contiguous; populations were much larger and thus less likely to be demographically independent. Altogether these results strongly argue against population structure being responsible for the signal we observe in the data.

It was generally believed that orang-utans were already at very low numbers a few decades ago. For instance, census estimates presented by Payne [6] suggesting that populations were still high across Sabah in the 1980s were considered optimistic and unrealistic. Our results thus represent the first independent confirmation that populations of orang-utans were still large a few decades ago, as suggested in [6], and recently demonstrated with aerial surveys in [32]. Although Payne's figures were based on limited and controversial demographic data, they appear to be indirectly confirmed by ongoing demographic, ecological, and behavioural surveys in the Lower Kinabatangan area [26] and in Sabah [32]. In the Kinabatangan region, population size estimates of around 200 individuals have been obtained for both S1 and S2 [26]. Given that census sizes are approximately three to ten times larger than effective sizes in vertebrates [17], these estimates are in good agreement with the posterior distribution of *N_0_* presented here. Ancrenaz et al. [26] also show non-equilibrium situations with densities that are unusually high for secondary forest environments, probably due to concentration in the remaining forest patches [26].

In conclusion, these results are the first to (1) demonstrate a clear genetic signature for a very recent orang-utan population decline, (2) quantify this decline, suggesting that it is at least one order of magnitude more important than classical census data have indicated, (3) support the originally controversial census estimates by Payne [6], and suggest that they were underestimates rather than overestimates, and (4) indicate, on purely genetic grounds and using equilibrium and non-equilibrium approaches, that current population size estimates (*N_0_*) are extremely low, implying that unless immediate action is taken, genetic drift will quickly eliminate the remaining genetic diversity in the fragmented forests of the Kinabatangan floodplain.

Our results strongly suggest that recent anthropogenic environmental changes are the main cause of the orang-utan population decline, that this decline is more recent and sharper than assumed, and that it has already led to the loss of significant genetic diversity, mostly through the loss of rare alleles [19,27]. It should be noted that our results do not necessarily go against the “overkill” hypothesis that appears to hold for many vertebrate species. In the case of orang-utans, the arrival of the first hunter-gatherers and farmers may well explain the current distribution of orang-utans and, in particular, their absence from islands where they are known to have been present. However, even if orang-utans from the Kinabatangan area suffered from early hunting, our analysis shows that the strength of the recent signal overcomes any earlier decrease. This is unprecedented and represents a new argument for the restoration of this unique ape's environment. In a region where between a third and half of the original forest area has been cleared in the last 30 y [8], and keeps being cleared, there is an urgent need to reconnect the remnant patches of the forests in order to increase the prospect of long-term survival of the species in the area. The uncertain future of orang-utans could still be improved, however. Our results also show that genetic diversity is still reasonably high (average heterozygosity, *H_e_* = 0.74) in the remaining Kinabatangan populations [29]. Ongoing demographic simulations that incorporate environmental and anthropogenic changes, including El Niño–caused droughts, and orang-utan persecution and hunting (M. W. Bruford, B. Goossens, L. Chikhi, I. Lackman-Ancrenaz, L. Ambu, and M. Ancrenaz, unpublished data) indicate that some of the populations analysed here are under high risk of becoming extinct within the next few decades. These simulations also show that the species could maintain its current level of variability and stabilise demographically if immediate steps are taken to halt anthropogenic habitat destruction, reconnect forest isolates where possible, and directly intervene where populations have become demographically unviable.

## Materials and Methods

### Study site

The Lower Kinabatangan floodplain is located in Eastern Sabah, Malaysia, and the Lower Kinabatangan Wildlife Sanctuary was gazetted in 2005 by the state government of Sabah. It encompasses ten forest blocks (termed “lots” 1–10, with Lot 10 divided into 10A–C; total area: 27,000 ha) which are linked to seven patches of protected forests (“Virgin Jungle Reserves”) to increase connectivity between forests fragments and create a corridor connecting the coastal mangrove swamps with dry land forests upriver (see Figure 1 in [29]).

### Sample collection

We collected shed hair from 176 orang-utan nests and faeces from 71 wild orang-utans encountered during boat surveys carried out between January and May 2001 alongside a 280-km river tract on the Kinabatangan River. Thirty-two faecal samples were also collected below fresh nests and were used instead of shed hairs collected in the nest. Therefore, of a total of 279 samples collected, 247 were selected for genetic analyses. The samples were assigned to nine sampling regions S1–S9 (see Figure 1 in [29]), corresponding to the lots described above, with the exception of samples in Lots 5 and 7, which were grouped into S5.

### DNA extraction and microsatellite amplification

We used a PCR buffer method described in [33] to extract DNA from shed hair (144 samples). To extract DNA from the 103 faecal samples, we used the QIAamp DNA Stool Mini Kit (Qiagen, Hilden, Germany) and followed a protocol for orang-utans detailed in [34] and [35]. All extractions were carried out in a Class I microbiological safety hood to avoid contamination. We amplified 14 human-derived microsatellite loci using a multiple-tube procedure conducted for each faecal extract according to [34] and [36]. The PCR amplification procedure is detailed in [29].

### Analysis of past demography

We used three different but complementary approaches to detect, quantify, and date the decline in orang-utan populations. The first approach uses summary statistics to detect population size changes, whereas the two other are full-likelihood Bayesian methods. They use the full allelic distribution in a Bayesian framework and allow the quantification of demographic parameters (see below for details). Both methods are implemented in the msvar programs available from M. Beaumont (http://www.rubic.rdg.ac.uk/~mab). The summary statistic–based method was applied to the whole Kinabatangan dataset (nine sampling units, *n* = 200 individuals, 14 loci). The second and third methods are highly computer intensive and were applied to two subsets of the data, S1 (*n* = 27) and S2 (*n* = 26), located on opposite sides of the 200 m–wide Kinabatangan River. Orang-utans from these populations are therefore demographically independent replicates. These two populations are among the three largest, with approximately 200 individuals each [26], and represent altogether approximately 20% of all individuals present in the Lower Kinabatangan floodplain [26]. For both methods, all runs had at least 10^9^ iterations with different thinning intervals (e.g., Tables S3–S6, last three columns). The runs were therefore approximately five to approximately 50 times longer than suggested by M. Beaumont in the *msvar* manuals.

### The *EWCL* (Ewens, Watterson, Cornuet, and Luikart) method

This method relies on the patterns of genetic diversity expected for a demographically stable population, using two summary statistics of the allelic frequency spectrum, namely the number of alleles *(n_A_)* and the expected heterozygosity *(H_e_)* [27,28]. Simulations were performed to obtain the distribution of *H_e_* conditional on *n_A_* and on the sample size for each population and locus. These *H_e_* values were then compared to those obtained from the real dataset. Three mutation models were used: the infinite allele model (IAM), the single stepwise model (SSM), and the two-phase model (TPM). Simulations were performed using Bottleneck software [24]. For the TPM, we allowed for 30% multi-step mutation events (a value of 0% corresponds to the SMM).

Departures from the null hypothesis can be explained by any departure from the model, including selection or population expansion or decline. Consistency across independent loci is unlikely to be caused by selection but rather by demographic events. This approach allowed us to detect population size changes and confirm that the signal was consistent across mutation models. However, the demographic event cannot be dated or quantified.

### The *Beaumont* method

This method [18] assumes that a stable population of size *N_1_* started to decrease (or increase) *t_a_* generations ago to the current population size, *N_0_*. The change in population size is assumed to be either linear or exponential, and mutations are assumed to occur under a stepwise mutation model (SMM), with a rate *θ* = 2*N_0_μ,* where μ is the locus mutation rate. Based on these assumptions, it is possible, using a Bayesian coalescent-based approach, to estimate the posterior probability distributions of (1) the rate of population size change *r* = *N_0_*/*N_1_,* (2) *t_f_* = *t_a_*/*N_0_* the time since the population started changing size, scaled by *N_0_,* and (3) *θ* = 2*N_0_μ*. The method uses a Markov Chain Monte Carlo (MCMC) approach to sample from the posterior distribution of these parameters. By using the information present in the full allelic distribution, this method allows the quantification of the population increase or decrease. However, *N_0_* and *N_1_* cannot be estimated independently. Similarly, the time since the population size change began can only be estimated as a time scaled by *N_0_,* which remains unknown. Thus, the population size change can be quantified, but it cannot be dated.

For each analysis at least three independent runs were performed using different parameter configurations and starting values. Note that the runs were all carried out with positive starting values of log*(r)* (Tables S3–S6). Such values correspond to a population expansion, rather than a population collapse. Thus, we avoided favouring regions of the parameter space corresponding to a population decline. Despite positive starting values, support for positive values was virtually zero.

In this method, rectangular prior distributions are assumed for log*(r)*, log*(θ),* and log*(t_f_)*. Wide bounds for these prior distributions were chosen (between 10^−5^ and 10^5^ on a natural log scale), so that posterior distributions would be little affected. In a few cases, some runs were duplicated as a test (e.g., Run 02 and Run 03 in Table S3), but we never found any discrepancy either between such runs or those starting from different values (Figure S1). All runs gave very similar posterior distributions for both S1 and S2 and for the two demographic models (Figure 1). Similar results were obtained for the other parameters of the model (not shown).

### The *Storz and Beaumont* method

This method [21] is an extension of Beaumont's method [18] and allows the estimation of *N_0_* and *N_1_,* rather than their ratio, and, crucially, also *T,* the time since the population change. In this model, prior distributions for *N_0_, N_1_, T,* and *θ,* are assumed to be log normal. The means and standard deviations of these log-normal distributions are themselves drawn from prior (or hyperprior) distributions. Means were drawn from normal distributions and standard deviations from normal distributions, truncated at zero.

Wide “uninformative” priors and multiple runs were used for both methods, as Table S7 shows, and variances for these prior distributions were large so as to affect posterior distributions as little as possible. Three runs (Runs 01, 02, and 03) were carried out by allowing *N_1_* to be either larger than or as large as *N_0_*. This had no effect on the posterior distribution (see Figure S2). Runs assuming that *N_1_* and *N_0_* were of the same size (i.e., no change in population size) were repeated several times as a test (Run 03 to Run 07 for S1 and Run 03 to Run 05 for S2) and provided exactly the same results as Runs 01 and 02 (Figure 2). The total number of iterations was always larger than 2 ×10^9^ and thinning values varied between 2 × 10^4^ and 5 × 10^4^.

The generation time of orang-utans is to a large extent unknown. In captive conditions, orang-utans can breed as early as 5 or 6 y old. We assumed a value of 8 y, but results can readily be extended to other values by multiplying them accordingly. Doubling this value does not change our conclusions (see Discussion).

## Supporting Information

Figure S1 Posterior Distributions for Log*(r)* Obtained for Independent MCMC Runs for S1 and S2 under the Models of Linear and Exponential Change Details of the runs can be found in Table S3.
(47 KB PDF).

Figure S2 Posterior Distributions Obtained for Independent MCMC Runs for S1 and S2 (A) Posterior distributions obtained for independent MCMC runs for S1. Details of the runs can be found in Table S7. The three sets of distributions correspond to *N_0_, N_1,_* and *T*.
(B) Same as (A) for S2.
(23 KB PDF).

Table S1 Results of the Bottleneck Analysis This table shows the results of the Bottleneck analyses for all the samples (S1–S9), for the three mutation models (IAM, infinite allele model; SMM, stepwise mutation model; and TPM, two-phase model), and for all loci (D5S1457, D5S1470, D1S550, D2S1326, D3S2459, D4S1627, D4S2408, D5S1505, D6S501, D13S321, D13S765, D12S375, D2S141, and D16S420). The figures represent the proportion of simulated datasets exhibiting apparent departures from equilibrium larger or as large as those observed in the data. Values below 0.05 thus indicate that the data observed exhibit a significant departure from equilibrium. One thousand simulations were done for each combination of locus/population/mutation model. The last column gives the result of the Wilcoxon test for population size change, across all loci for all samples and for all mutation models. All tests, except one, are highly significant whichever mutation model or population is analysed. The only test that is not highly significant is SMM for S2. This test is borderline, with an estimated probability of 0.052. This means that S2 is one of the populations for which the signal for population decrease is lowest. S2 thus represents a conservative estimate for population decrease in the following analyses. Details of the mutation models can be found in [28] and the Bottleneck manual.
(90 KB DOC).

Table S2 Population Substructure Vitalis and Couvet's method [31] was applied to the data. This method aims at separating the effect of migration rates and genetic drift in creating genetic structure in populations. Using this method, it is possible to estimate migration rates and a parameter *F,* which is a measure of population substructure. Most values are low and indicate low or moderate levels of population structure. The only exceptions are S3 and S7, with medium values of 0.11. Interestingly, S7 is the smallest population, with a census size of only 22 individuals. When all samples from both riversides are grouped together and the analysis is carried out again, the *F* values decrease below 0.05 (0.029 and 0.044).
(29 KB DOC).

Table S3 Details of the MCMC Runs for S1 under the Model of Linear Size Change Starting values are given in the first three columns. The range corresponds to the range of the rectangular prior distributions for all the parameters. See Beaumont [18] for details.
(30 KB DOC).

Table S4 Details of the MCMC Runs for S1 under the Model of Exponential Size Change Same as Table S3.
(26 KB DOC).

Table S5 Details of the MCMC Runs for S2 under the Model of Linear Size Change Same as Table S3.
(29 KB DOC).

Table S6 Details of the MCMC Runs for S2 under the Model of Exponential Size Change Same as Table S3.
(29 KB DOC).

Table S7 Parameters for the *Storz and Beaumont* Method Runs Columns 1 to 4: The two figures correspond to the starting values of the mean and variance of the corresponding parameters. For instance, across all runs, starting values for ancestral and current population sizes were assumed to be similar and large (*N_0_* ~*N_1_* ~10^4^). These values are updated during the MCMC, using hyperpriors defined by columns 5 to 8.
Columns 5 to 8: The four figures correspond to the hyperprior means and variances for the means and variances for the corresponding parameters.
For instance, for Run 01, *N_1_* is sampled from a prior log-normal distribution with a mean that is itself drawn from a normal distribution with mean 5 and variance 3 (on a log scale). The starting value for the mean is 4, as column 2 shows. The variance is drawn from a truncated normal distribution, but the mean of the variance is 0, and its variance 0.5. The starting value for the mean variance in the first run is 1, as shown in column 2.
The only differences between Runs 03–07 are that different thinning values and different lengths of the total MCMC were used. They all gave similar results, as can be seen in Figure 2. Runs 06 and 07 were carried out for S1 but not S2, due to the very good convergence already obtained with the other runs. Thus S2 has three repetitions of Run 03 instead of five.
(33 KB DOC).
