# Supplementary material for: Genetic Signature of Anthropogenic Population Collapse in Orang-utans
Source: PLoS Biol. 2006 Jan 24;4(2):e25. doi: 10.1371/journal.pbio.0040025 (PMC1334199; doi:10.1371/journal.pbio.0040025)
Supplement: Supplementary file 1 [file pbio.0040025.sg001.pdf]

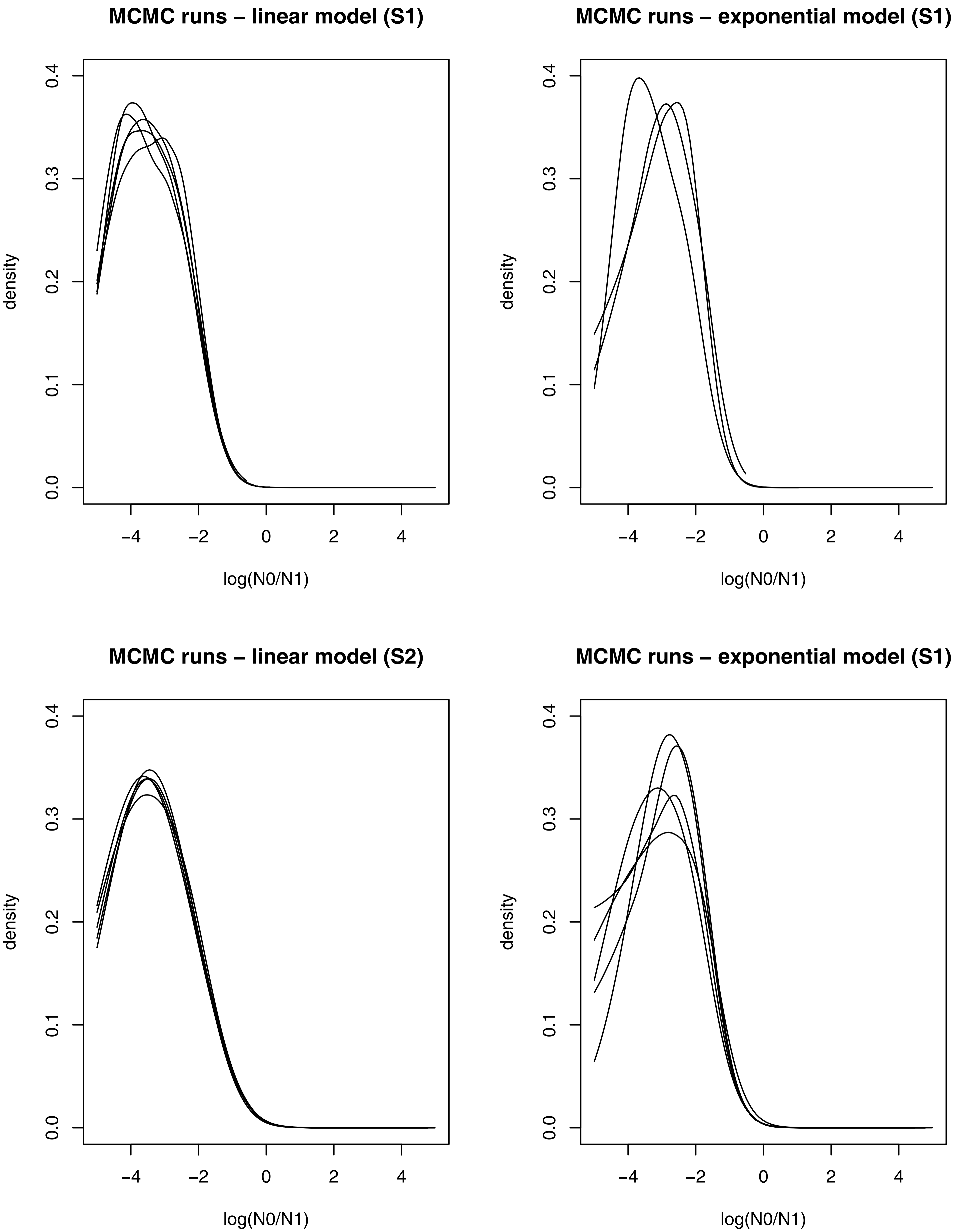
MCMC runs – linear model (S1)
MCMC runs – exponential model (S1)
MCMC runs – linear model (S2)
MCMC runs – exponential model (S1)
density
0.0
0.1
0.2
0.3
0.4
log(N0/N1)
−4
−2
0
2
4
