# Supplementary material for: Genetic Signature of Anthropogenic Population Collapse in Orang-utans
Source: PLoS Biol. 2006 Jan 24;4(2):e25. doi: 10.1371/journal.pbio.0040025 (PMC1334199; doi:10.1371/journal.pbio.0040025)
Supplement: Supplementary file 2 [file pbio.0040025.sg002.pdf]

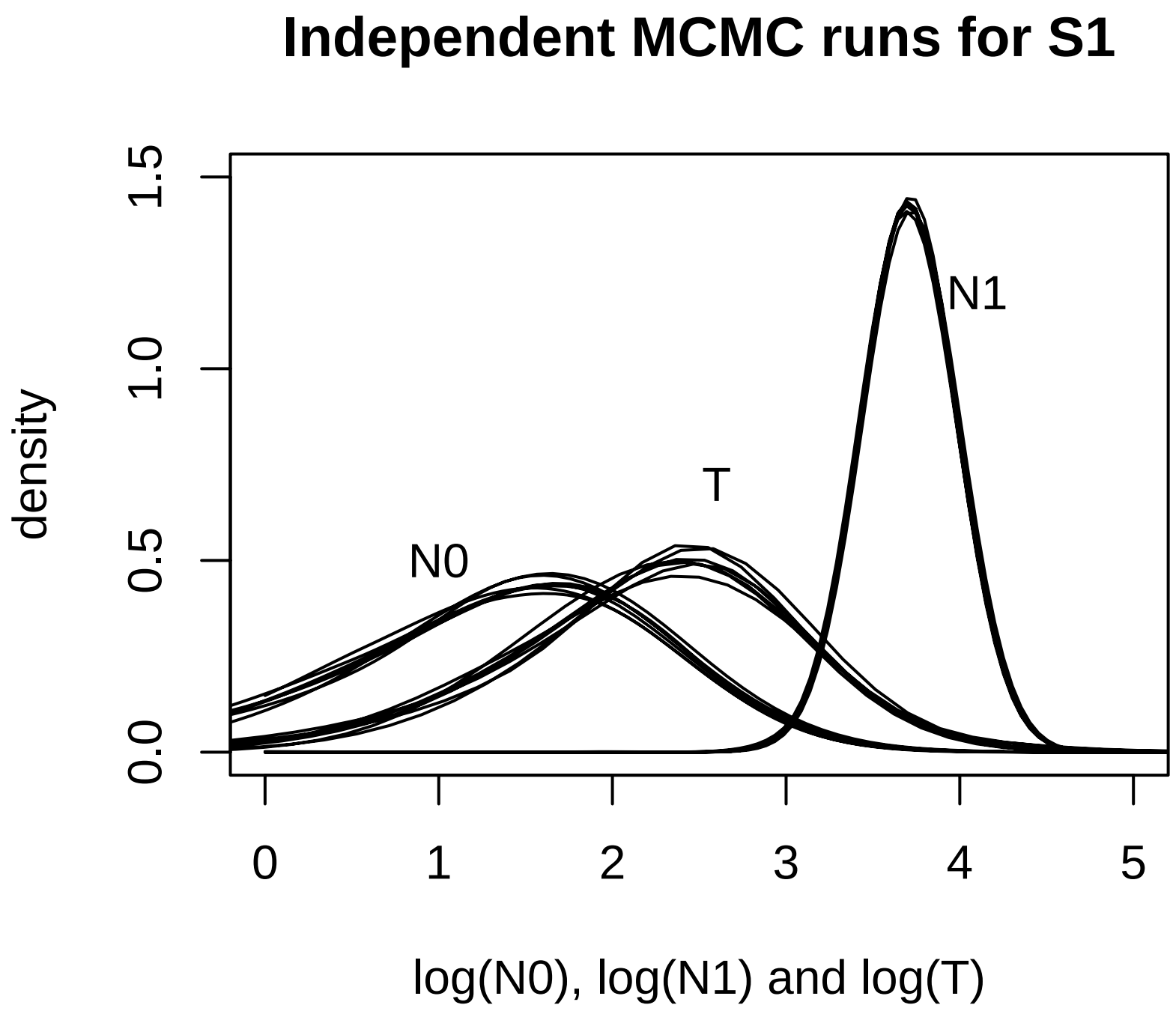
Independent MCMC runs for S1
density
0.0
0.5
1.0
1.5
N0
T
N1
0
1
2
3
4
5
log(N0), log(N1) and log(T)

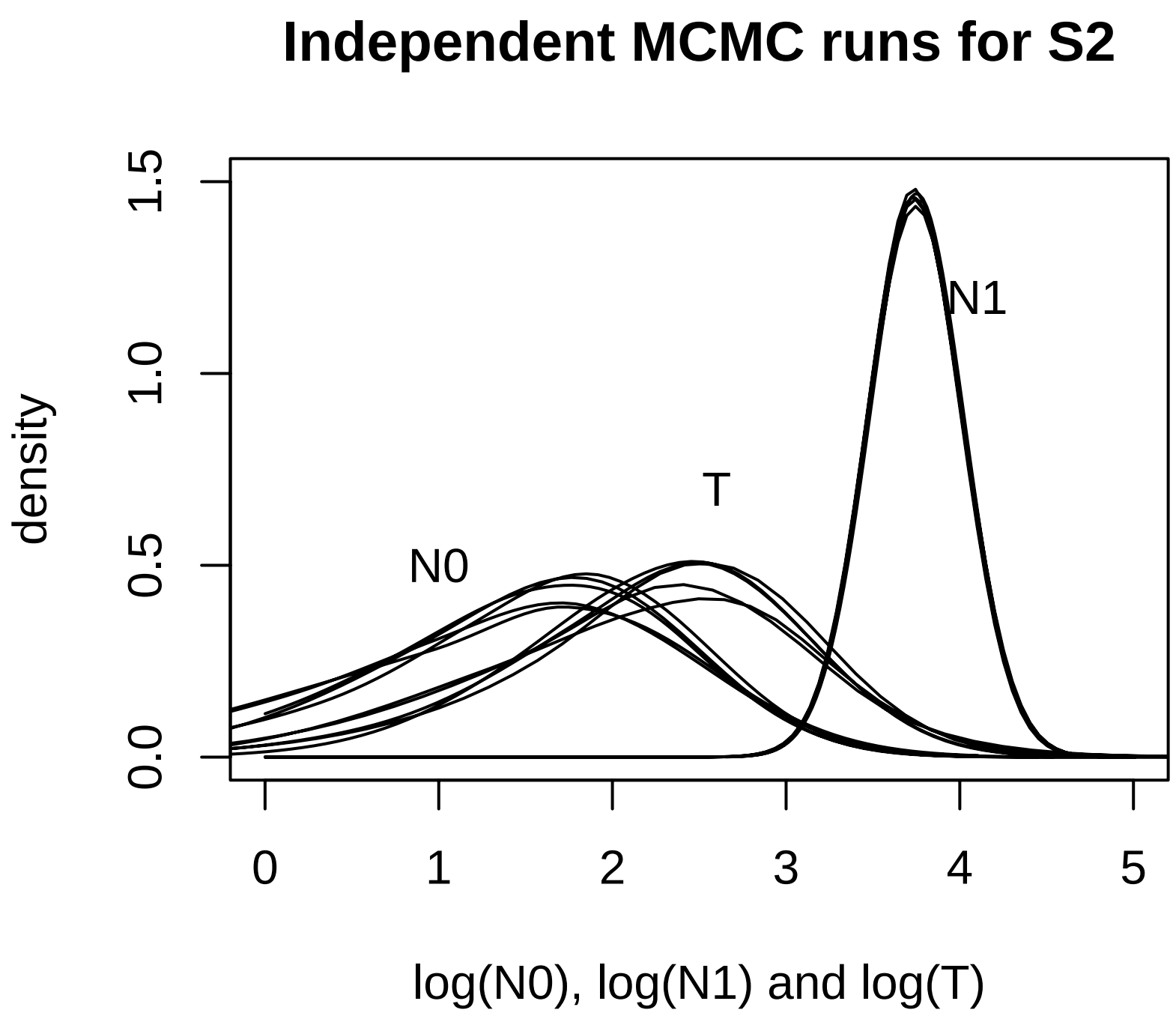
Independent MCMC runs for S2
density
0.0
0.5
1.0
1.5
N0
T
N1
0
1
2
3
4
5
log(N0), log(N1) and log(T)
