# Supplementary material for: Genetic Signature of Anthropogenic Population Collapse in Orang-utans
Source: PLoS Biol. 2006 Jan 24;4(2):e25. doi: 10.1371/journal.pbio.0040025 (PMC1334199; doi:10.1371/journal.pbio.0040025)
Supplement: Supplementary file 3 [file pbio.0040025.st001.doc]

| Table S1 | |  |  |  |  |  |  |  |  |  |  |  |  |  |  |  |
| --- | --- | --- | --- | --- | --- | --- | --- | --- | --- | --- | --- | --- | --- | --- | --- | --- |
|  |  |  |  |  |  |  |  | Loci | |  |  |  |  |  |  |  |
| Population | Mutation model | D5S1457 | D5S1470 | D1S550 | D2S1326 | D3S2459 | D4S1627 | D4S2408 | D5S1505 | D6S501 | D13S321 | D13S765 | D12S375 | D2S141 | D16S420 | Wilcoxon |
| S1 | I.A.M. | 0,022 | 0,045 | 0,031 | 0,056 | 0,356 | 0,285 | 0,118 | 0,007 | 0,157 | 0,000 | 0,157 | 0,030 | 0,075 | 0,068 | 0,00003 |
|  | T.P.M. | 0,054 | 0,087 | 0,072 | 0,088 | 0,439 | 0,470 | 0,203 | 0,022 | 0,242 | 0,001 | 0,296 | 0,055 | 0,135 | 0,133 | 0,00003 |
|  | S.M.M. | 0,113 | 0,230 | 0,177 | 0,220 | 0,225 | 0,291 | 0,371 | 0,044 | 0,342 | 0,012 | 0,474 | 0,086 | 0,270 | 0,269 | 0,00058 |
| S2 | I.A.M. | 0,073 | 0,265 | 0,059 | 0,125 | 0,008 | 0,328 | 0,202 | 0,008 | 0,220 | 0,008 | 0,494 | 0,245 | 0,086 | 0,091 | 0,00003 |
|  | T.P.M. | 0,185 | 0,445 | 0,119 | 0,197 | 0,020 | 0,473 | 0,339 | 0,035 | 0,368 | 0,016 | 0,261 | 0,415 | 0,122 | 0,201 | 0,00021 |
|  | S.M.M. | 0,405 | 0,301 | 0,274 | 0,487 | 0,053 | 0,229 | 0,501 | 0,113 | 0,475 | 0,045 | 0,093 | 0,421 | 0,199 | 0,343 | 0,05200 |
| S3 | I.A.M. | 0,044 | 0,003 | 0,188 | 0,080 | 0,238 | 0,279 | 0,193 | 0,021 | 0,286 | 0,210 | 0,236 | 0,112 | 0,019 | 0,060 | 0,00003 |
|  | T.P.M. | 0,069 | 0,005 | 0,276 | 0,159 | 0,372 | 0,442 | 0,276 | 0,044 | 0,435 | 0,326 | 0,399 | 0,185 | 0,057 | 0,059 | 0,00003 |
|  | S.M.M. | 0,103 | 0,008 | 0,446 | 0,353 | 0,445 | 0,320 | 0,411 | 0,088 | 0,440 | 0,488 | 0,381 | 0,249 | 0,127 | 0,106 | 0,00043 |
| S4 | I.A.M. | 0,127 | 0,471 | 0,245 | 0,084 | 0,067 | 0,030 | 0,016 | 0,013 | 0,328 | 0,005 | 0,035 | 0,284 | 0,000 | 0,003 | 0,00003 |
|  | T.P.M. | 0,240 | 0,306 | 0,388 | 0,116 | 0,107 | 0,068 | 0,038 | 0,034 | 0,416 | 0,016 | 0,071 | 0,460 | 0,000 | 0,005 | 0,00015 |
|  | S.M.M. | 0,415 | 0,116 | 0,326 | 0,222 | 0,224 | 0,151 | 0,055 | 0,098 | 0,189 | 0,031 | 0,132 | 0,315 | 0,001 | 0,013 | 0,01227 |
| S5 | I.A.M. | 0,017 | 0,047 | 0,007 | 0,263 | 0,067 | 0,378 | 0,037 | 0,011 | 0,340 | 0,003 | 0,185 | 0,087 | 0,022 | 0,002 | 0,00003 |
|  | T.P.M. | 0,044 | 0,112 | 0,008 | 0,433 | 0,153 | 0,409 | 0,060 | 0,026 | 0,446 | 0,014 | 0,236 | 0,127 | 0,039 | 0,002 | 0,00003 |
|  | S.M.M. | 0,112 | 0,251 | 0,045 | 0,281 | 0,297 | 0,165 | 0,072 | 0,062 | 0,185 | 0,023 | 0,510 | 0,269 | 0,075 | 0,005 | 0,00830 |
| S6 | I.A.M. | 0,059 | 0,053 | 0,011 | 0,003 | 0,061 | 0,024 | 0,033 | 0,001 | 0,184 | 0,002 | 0,116 | 0,077 | 0,020 | 0,043 | 0,00003 |
|  | T.P.M. | 0,118 | 0,125 | 0,023 | 0,004 | 0,122 | 0,035 | 0,073 | 0,009 | 0,288 | 0,005 | 0,234 | 0,119 | 0,044 | 0,096 | 0,00003 |
|  | S.M.M. | 0,277 | 0,283 | 0,071 | 0,010 | 0,303 | 0,083 | 0,147 | 0,024 | 0,475 | 0,018 | 0,483 | 0,244 | 0,115 | 0,238 | 0,00003 |
| S7 | I.A.M. | 0,289 | 0,343 | 0,170 | 0,656 | 0,040 | 0,203 | 0,204 | 0,575 | 0,406 | 0,556 | 0,382 | 0,505 | 0,271 | 0,284 | 0,00043 |
|  | T.P.M. | 0,371 | 0,231 | 0,247 | 0,416 | 0,056 | 0,259 | 0,257 | 0,650 | 0,506 | 0,679 | 0,440 | 0,594 | 0,407 | 0,367 | 0,00671 |
|  | S.M.M. | 0,483 | 0,202 | 0,309 | 0,321 | 0,070 | 0,315 | 0,279 | 0,438 | 0,537 | 0,419 | 0,4700 | 0,677 | 0,467 | 0,463 | 0,03925 |
| S8 | I.A.M. | 0,368 | 0,001 | 0,412 | 0,002 | 0,019 | 0,025 | 0,140 | 0,000 | 0,448 | 0,001 | 0,085 | 0,017 | 0,002 | 0,058 | 0,00003 |
|  | T.P.M. | 0,405 | 0,001 | 0,395 | 0,002 | 0,036 | 0,042 | 0,222 | 0,000 | 0,371 | 0,009 | 0,191 | 0,026 | 0,006 | 0,126 | 0,00031 |
|  | S.M.M. | 0,166 | 0,004 | 0,238 | 0,005 | 0,076 | 0,074 | 0,353 | 0,003 | 0,174 | 0,018 | 0,395 | 0,031 | 0,010 | 0,326 | 0,00671 |
| S9 | I.A.M. | 0,001 | 0,103 | 0,010 | 0,204 | 0,130 | 0,012 | 0,071 | 0,106 | 0,278 | 0,000 | 0,219 | 0,036 | 0,027 | 0,000 | 0,00009 |
|  | T.P.M. | 0,007 | 0,169 | 0,010 | 0,331 | 0,233 | 0,028 | 0,120 | 0,194 | 0,417 | 0,001 | 0,109 | 0,085 | 0,060 | 0,000 | 0,00214 |
|  | S.M.M. | 0,025 | 0,333 | 0,023 | 0,476 | 0,446 | 0,051 | 0,232 | 0,373 | 0,370 | 0,003 | 0,030 | 0,119 | 0,129 | 0,000 | 0,00830 |
