# Supplementary material for: Genetic Signature of Anthropogenic Population Collapse in Orang-utans
Source: PLoS Biol. 2006 Jan 24;4(2):e25. doi: 10.1371/journal.pbio.0040025 (PMC1334199; doi:10.1371/journal.pbio.0040025)
Supplement: Supplementary file 7 [file pbio.0040025.st005.doc]

##### Table S5

| S2 (exp.) | log(*θ*) | log(*r*) | log(*t_f_*) | range | thinning | steps | iterations |
| --- | --- | --- | --- | --- | --- | --- | --- |
| Run_01 | 100 | 1000 | 10 | 10^-5^-10^5^ | 5 10^5^ | 2 10^4^ | 10^10^ |
| Run_02 | 1 | 10 | 1000 | 10^-5^-10^5^ | 10^6^ | 5 10^4^ | 5 10^10^ |
| Run_03 | 10 | 10 | 10 | 10^-5^-10^5^ | 5 10^5^ | 2 10^4^ | 10^10^ |
| Run_04 | 10 | 100 | 100 | 10^-5^-10^5^ | 5 10^5^ | 2 10^4^ | 10^10^ |
| Run_05 | 1000 | 1 | 10 | 10^-5^-10^5^ | 5 10^5^ | 2 10^4^ | 10^10^ |
