# Supplementary material for: Genetic Signature of Anthropogenic Population Collapse in Orang-utans
Source: PLoS Biol. 2006 Jan 24;4(2):e25. doi: 10.1371/journal.pbio.0040025 (PMC1334199; doi:10.1371/journal.pbio.0040025)
Supplement: Supplementary file 8 [file pbio.0040025.st006.doc]

Table S6

| Runs | log(*N_0_*) | log(*N_1_*) | log(*θ*) | log(*T*) | log(*N_0_*) | log(*N_1_*) | log(*θ*) | log(*T*) |
| --- | --- | --- | --- | --- | --- | --- | --- | --- |
| Run 01 | 4 1 | 4 1 | -3.5 1 | 5 1 | 3 2 0 0.5 | 5 3 0 0.5 | -3.5 0.25 0 0.5 | 5 3 0 0.5 |
| Run 02 | 4 1 | 4 1 | -3.5 1 | 5 1 | 3 2 0 0.5 | 4 3 0 0.5 | -3.5 0.25 0 0.5 | 5 3 0 0.5 |
| Run 03 | 4 1 | 4 1 | -3.5 1 | 5 1 | 3 2 0 0.5 | 3 3 0 0.5 | -3.5 0.25 0 0.5 | 5 3 0 0.5 |
| Run 04 | 4 1 | 4 1 | -3.5 1 | 5 1 | 3 2 0 0.5 | 3 3 0 0.5 | -3.5 0.25 0 0.5 | 5 3 0 0.5 |
| Run 05 | 4 1 | 4 1 | -3.5 1 | 5 1 | 3 2 0 0.5 | 3 3 0 0.5 | -3.5 0.25 0 0.5 | 5 3 0 0.5 |
| Run 06 | 4 1 | 4 1 | -3.5 1 | 5 1 | 3 2 0 0.5 | 3 3 0 0.5 | -3.5 0.25 0 0.5 | 5 3 0 0.5 |
| Run 07 | 4 1 | 4 1 | -3.5 1 | 5 1 | 3 2 0 0.5 | 3 3 0 0.5 | -3.5 0.25 0 0.5 | 5 3 0 0.5 |
