# Supplementary material for: Genetic Signature of Anthropogenic Population Collapse in Orang-utans
Source: PLoS Biol. 2006 Jan 24;4(2):e25. doi: 10.1371/journal.pbio.0040025 (PMC1334199; doi:10.1371/journal.pbio.0040025)
Supplement: Supplementary file 9 [file pbio.0040025.st007.doc]

Table S7

| Population | F | 95% interval | |
| --- | --- | --- | --- |
| S1 | 0,058 | 0,048 | 0,068 |
| S2 | 0,075 | 0,064 | 0,085 |
| S3 | 0,110 | 0,097 | 0,123 |
| S4 | 0,012 | 0,000 | 0,023 |
| S5 | 0,017 | 0,011 | 0,023 |
| S6 | 0,014 | 0,010 | 0,018 |
| S7 | 0,118 | 0,102 | 0,135 |
| S8 | 0,021 | 0,011 | 0,034 |
| S9 | -0,005 | -0,016 | 0,007 |
